# Specific Interaction of Gαi3 with the Oa1 G-Protein Coupled Receptor Controls the Size and Density of Melanosomes in Retinal Pigment Epithelium

**DOI:** 10.1371/journal.pone.0024376

**Published:** 2011-09-08

**Authors:** Alejandra Young, Meisheng Jiang, Ying Wang, Novruz B. Ahmedli, John Ramirez, Benjamin E. Reese, Lutz Birnbaumer, Debora B. Farber

**Affiliations:** 1 Jules Stein Eye Institute, University of California Los Angeles, Los Angeles, California, United States of America; 2 Molecular Biology Institute, University of California Los Angeles, Los Angeles, California, United States of America; 3 Department of Molecular and Medical Pharmacology, University of California Los Angeles, Los Angeles, California, United States of America; 4 Department of Psychological and Brain Sciences, University of California Santa Barbara, Santa Barbara, California, United States of America; 5 Laboratory of Neurobiology, Division of Intramural Research, National Institute of Environmental Health Sciences, National Institutes of Health, Research Triangle Park, North Carolina, United States of America; Harvard Medical School, United States of America

## Abstract

**Background:**

Ocular albinism type 1, an X-linked disease characterized by the presence of enlarged melanosomes in the retinal pigment epithelium (RPE) and abnormal crossing of axons at the optic chiasm, is caused by mutations in the *OA1* gene. The protein product of this gene is a G-protein-coupled receptor (GPCR) localized in RPE melanosomes. The *Oa1-/-* mouse model of ocular albinism reproduces the human disease. Oa1 has been shown to immunoprecipitate with the Gαi subunit of heterotrimeric G proteins from human skin melanocytes. However, the Gαi subfamily has three highly homologous members, Gαi1, Gαi2 and Gαi3 and it is possible that one or more of them partners with Oa1. We had previously shown by *in-vivo* studies that *G*α*i3-/-* and *Oa1-/-* mice have similar RPE phenotype and decussation patterns. In this paper we analyze the specificity of the Oa1-Gαi interaction.

**Methodology:**

By using the genetic mouse models *Gαi1-/-, Gαi2-/-, Gαi3-/-* and the double knockout *G*α*i1-/-, G*α*i3-/-* that lack functional Gαi1, Gαi2, Gαi3, or both Gαi1 and Gαi3 proteins, respectively, we show that Gαi3 is critical for the maintenance of a normal melanosomal phenotype and that its absence is associated with changes in melanosomal size and density. GST-pull-down and immunoprecipitation assays conclusively demonstrate that Gαi3 is the only Gαi that binds to Oa1. Western blots show that Gαi3 expression is barely detectable in the *Oa1-/-* RPE, strongly supporting a previously unsuspected role for Gαi3 in melanosomal biogenesis.

**Conclusion:**

Our results identify the Oa1 transducer Gαi3 as the first downstream component in the Oa1 signaling pathway.

## Introduction

Hypopigmentation mutations affecting melanin synthesis or melanosomal biogenesis in the retinal pigment epithelium (RPE) of mammals are known to have profound effects on the developing retina and visual pathways, including abnormal crossing of the optic axons, nystagmus, strabismus, foveal hypoplasia, and reduced visual acuity [1]. Two forms of albinism are commonly recognized: oculocutaneous albinism (OCA), in which neither the eye nor the skin or hair are pigmented and ocular albinism (OA), which affects primarily the eye pigmentation.

Ocular albinism type 1 (*OA1*, also called Nettleship-Falls type), is the most common form of ocular albinism. It has an estimated prevalence of 1/50,000 in the general population of the United States [2]. Although the cutaneous manifestations of *OA1* are very mild, affected patients present abnormal macromelanosomes in both the RPE and skin [3]. In contrast to other forms of albinism, melanin is not dramatically reduced in *OA1*; in fact, this disease is characterized by the unusual coexistence of the typical albino visual defects with a substantial amount of melanin in the eyes [4]. Different types of mutations in the *OA1* gene have been associated with ocular albinism type 1 (http://albinismdb.med.umn.edu/oa1mut.html#mutations). OA1, the protein product of the *OA1* gene, is a G protein-coupled receptor localized to RPE melanosomal membranes and the initiator of the observed abnormal visual phenotype in ocular albinism. The position of OA1 within these membranes, with its N-terminal towards the lumen of the melanosome and C-terminal towards the cytoplasm, suggests that it may function as a novel intracellular GPCR activated by the binding of a melanosomal ligand. This ligand could thus regulate melanosomal biogenesis through activation of specific G-proteins found in the RPE cytoplasm [5]. In addition, this localization of OA1 is consistent with its proposed role as a stop signal for melanosome overgrowth during melanogenesis [6], [7] and could explain the changes in RPE phenotype observed in ocular albinism: mutations or deletions in *OA1* producing a non functional OA1 protein would allow a continuous vesicular traffic of membrane proteins to melanosomes resulting in the formation of macromelanosomes [3].

It has been shown that the endogenous OA1 from human melanocyte extracts co-immunoprecipitates mainly with the alpha subunits of the Gi subfamily of heterotrimeric G-proteins [8]. The Gαi subfamily comprises three closely related members, Gαi1, Gαi2, and Gαi3, which share 85 to 95% amino acid sequence identity and partial overlapping expression patterns. In addition to their established function in signal transduction across the plasma membrane, these heterotrimeric Gi proteins localize to intracellular membranes and have been implicated in the regulation of membrane trafficking and fusion events along the secretory and endocytic pathways, such as vesicle formation by the endoplasmic reticulum, the Golgi/secretory pathway, and vesicle trafficking and fusion [9], [10], [11]. We have recently shown that mouse Oa1 and Gαi3 play an important role in the determination of melanosomal size and density and that both signal in the same pathway to regulate axonal guidance at the optic chiasm [12]. In the present study, we used Gαi knockout mouse models to investigate whether the other two members of the Gαi family of proteins, Gαi1 and Gαi2, are involved in the regulation of size and density of the RPE melanosomes. We also studied the effects of the loss of both Gαi1 and Gαi3 using the corresponding double knockout mice (heretofore called DKO). In addition, we analyzed the specific interaction of each Gαi protein with Oa1 in *in-vitro* immunoprecipitation and GST-pull-down experiments. For the latter, two polypeptides corresponding to the Oa1 third intracellular loop (i3) and carboxy-terminal tail (CT), the two regions that have been shown to play a critical role as selectivity determinants in receptor–G-protein interactions [13], [14], were tested. Furthermore, we investigated the expression and cellular response of Gαi proteins in the RPE of *Oa1-/-* mice using Western blots and ADP-ribosylation. Altogether, our *in-vivo* and *in-vitro* data demonstrate a previously unknown role of Gαi3 as the specific protein downstream of Oa1 that regulates the size and density of RPE melanosomes.

## Results

### Size, density and morphology of RPE melanosomes

**Figure 1:**
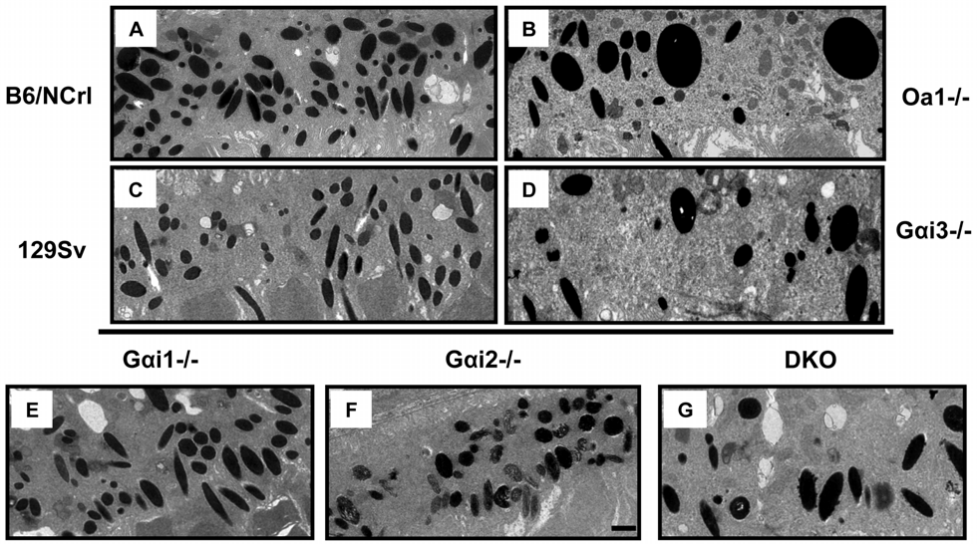
Appearance of RPE melanosomes from *Gαi1-/-, Gαi2-/-* and DKO *Gαi1-/-, Gαi3-/-* mice. RPEs from *Oa1-/-* (B) and their control B6/NCrl (A) mice and *Gαi3-/-* (D) and their control 129 Sv (C) mice have been published before [12] and are shown for comparison. The ultrathin RPE sections show that *Gαi1-/-* (E) and *Gαi2 -/- (F)* mice do not have macromelanosomes as those present in *Oa1-/-* (B), *Gαi3-/- (D)* and DKO *Gαi1-/-, Gαi3-/- (G)* mice. Electron micrographs of ultrathin sections, 16,000× magnification; scale bars for all micrographs, 01 µm, is shown only in (F).

To investigate the involvement of Gαi proteins in the regulation of size and density of RPE melanosomes, we analyzed electron-micrographs of RPE melanosomes from each *Gαi1-/-, Gαi2-/-, Gαi3-/-* and DKO mice, using 129 Sv wild-type mice as controls, as discussed in Materials and Methods. We had previously shown that *Gαi3-/-* mice, like *Oa1-/-* mice, have larger RPE melanosomes than those of control mice, and that they each have reduced density of melanosomes [12] (Figures 1A, B, C and D). Notably, the two background strains for these knock-out mice (129 Sv and C57Bl/6NCrl, hereafter B6/NCrl) differ substantially in melanosome size and density, making clear the need for appropriate controls when considering these features of the RPE. *Oa1-/-* macromelanosomes are conspicuously larger than *Gαi3-/-* macromelanosomes, but the latter are still significantly larger than those found in the 129 Sv control mice [12]. Our current electron microscopy results compare the relative roles of Gαi1 and Gαi2 to that of Gαi3 upon melanosomal size, density and shape, and assess the effects of combining the loss of two of them in a double knockout mouse.

We first examined the size of melanosomes in the RPE of these different Gαi knockout mice. The RPE of *Gαi1*-/- mice shows melanosomes that appear slightly enlarged (Figure 1E), while melanosomes in *Gαi2*-/- mice appear no different (Figure 1F), when compared with the melanosomes of 129 Sv mice (Figure 1C). By contrast, the RPE in DKO mice appears to contain enlarged melanosomes relative to those in 129 Sv mice, being comparable in size to those in the RPE of *Gαi3-/-* mice (Figure 1D). In order to quantify these differences, we measured melanosomal size, and determined the relative frequency of larger, macro-melanosomes (>5000 nm^2^) within the RPE of the different *Gαi*-/- mice. A total of 2501 129 Sv, 1285 G*αi1-/-*, 1609 G*αi2-/-*, 1115 G*αi3-/-* and 1997 DKO melanosomes were sampled.

**Figure 2:**
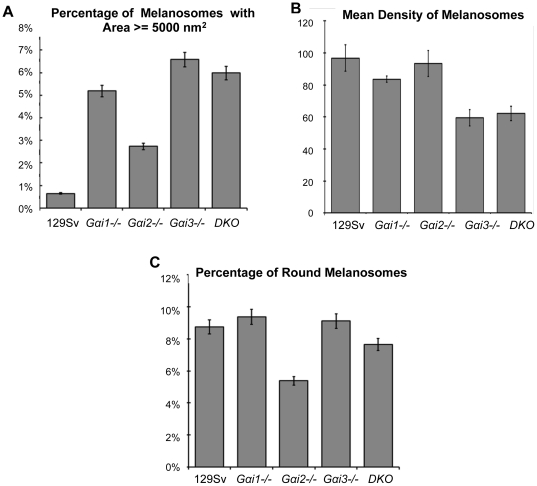
RPE melanosomal size, density and morphology of all *Gαi-/- mice* compared to control 129 Sv mice. (**A**) Percentage of melanosomes larger than 5000 nm^2^ in each of the mouse lines analyzed. The *Gαi3-/-* mice have the highest percentage of melanosomes larger than 5000 nm^2^ in the RPE. (**B**) Mean density of melanosomes: The *Gαi3-/-* group had the lowest mean number of melanosomes per RPE area (59±5.2), and the wild-type and *Gαi2-/-* groups had the largest mean density of melanosomes (96.8±8.2 and 93.4±8.1, respectively). (**C**) Percentage of round melanosomes: The *Gαi1-/-* and *Gαi3-/-* groups have the larger number of round melanosomes (9.37±0.77 and 9.12±1.85 respectively), followed by the 129 Sv wild-type (8.75%±0.25), DKO (7.65%±0.31) and *Gαi2-/-* (5.39%±0.37).

Comparison of the area of melanosomes from each mouse line (Figure 2A) shows that the frequency of larger melanosomes significantly differs between the groups, (ANOVA, p<0.05). *Gαi3-/-* RPEs have the highest percentage of melanosomes larger than 5000 nm^2^ (6.58%±1.47), and post-hoc Tukey tests confirmed that this group is significantly larger than the 129 Sv control retinas (p<0.05). Thus, loss of only Gai3 yielded a significant increase in the presence of larger melanosomes. Combining the loss of Gαi1 with Gαi3 proteins in the DKO mice certainly did not worsen the abnormal morphology of the *Gαi3-/-* RPE melanosomes. Together, these results suggest that only Gαi3 has a function in the determination of the size of these organelles.

Second, we evaluated melanosome density (number of melanosomes/RPE µm^2^) in the RPE of all the *Gαi*-/- and their 129 Sv control mice (Figure 2B). While melanosomal density need not be inversely related to melanosome size, we previously found that it was in both *G*α*i3-/-* and in *Oa1-/-* mice [12], and so extended these analyses to the present knock-out mice. *Gαi1-/-* and *Gαi2-/-* mice showed comparable densities to those of 129 Sv mice, while *Gαi3*-/- mice had a large reduction (37.3%) in melanosomal density when compared to 129 Sv mice. DKO mice also showed a comparable effect (a 34.6% reduction). Statistical analysis confirmed an effect of group (ANOVA, p<0.05), and post-hoc Tukey tests confirmed that *Gαi3-/-* and DKO are significantly different from both 129 Sv and *Gαi2*-/- mice (p<0.05). These results and the fact that the loss of Gαi1 alone had no significant effect on either melanosome size or density leads us to conclude that of the three heterotrimeric Gαi proteins, Gαi3 may be the endogenous downstream protein in the Oa1 signaling cascade that controls melanosomal size and density.

Third, we compared the morphology of the melanosomes in the RPE of the different *Gαi*-/- mice with that of their control mice, 129 Sv, to determine the percentage of melanosomes with a round shape. We classified all melanosomes according to their sphericity level, as described in Materials and Methods. Comparisons of 129 Sv with each *Gαi-/-* mouse studied showed that all animals except for *Gαi2-/-* have a similar percentage of RPE melanosomes that are round: 129 Sv 8.75%±0.25, *Gαi1-/-* 9.37%±0.77, *Gαi2-/-* 5.39%±0.37, *Gαi3-/-* 9.12%±1.85, and DKO *7.65*%±0.31. One-way analysis of variance, however, failed to reveal any effect of group despite the appearance of fewer round melanosomes in *Gαi2-/-* (Figure 2C).

### Analysis of retinal function in all *Gαi-/-* mice by electroretinography

**Figure 3:**
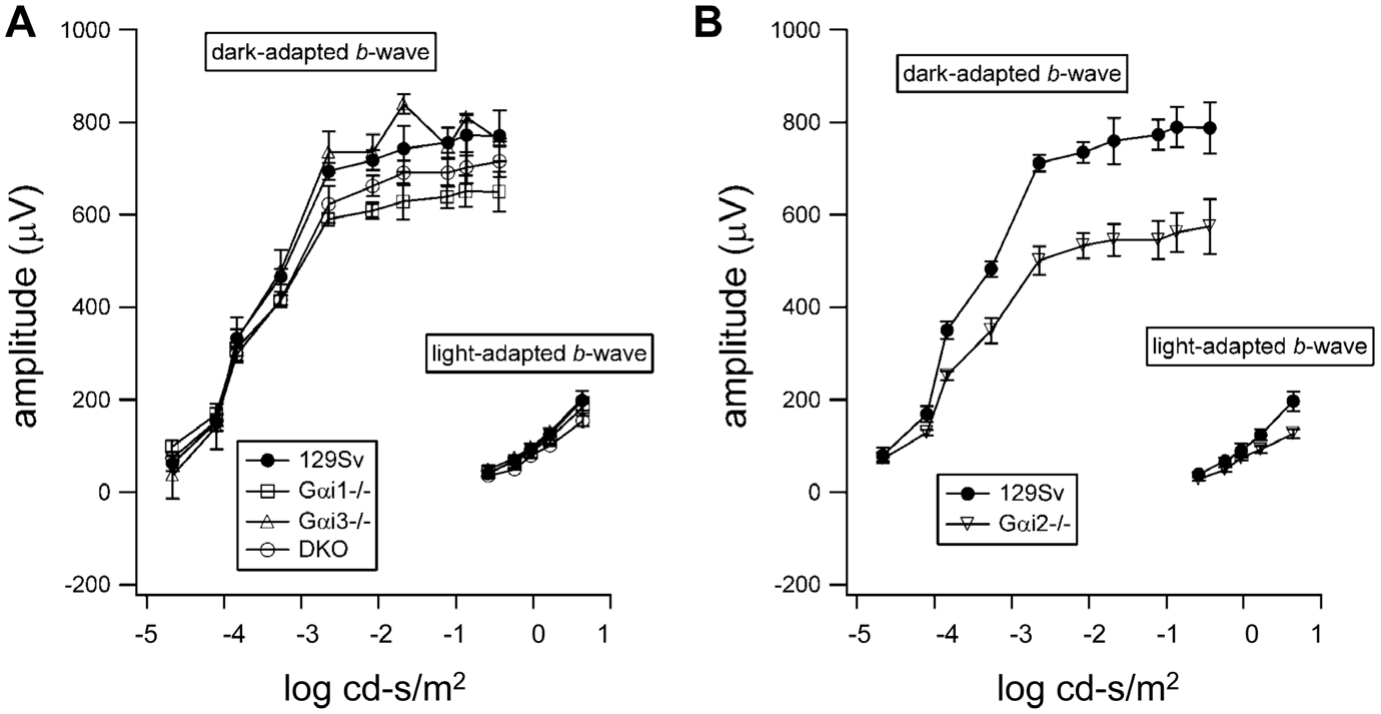
Representative ERG responses of *Gαi1-/-, Gαi2-/-, Gαi3-/-* and DKO mice. Standardized ERG was performed in experimental and control mice in two different sessions. **A**) ERGs from wild-type, *Gαi1-/-, Gαi3-/-* and DKO mice. **B**) ERGs from wild-type and *Gαi2-/-* mice.

ERG responses obtained from129 Sv, *Gαi1-/-, Gαi3*-/- and DKO mice are summarized in Figure 3A and from 129 Sv and *Gαi2*-/- mice in Figure 3B. In each panel, mean response amplitudes (±1sd) are plotted against stimulus intensity (left to right). The retinal function of *Gαi1-/-, Gαi3-/-* and DKO mice, as judged by the electroretinograms, appears to be not significantly different and within normal limits from control retinas (Figure 3A). Curiously, the ERGs of the *Gαi2-/-* mice show a reduced b-wave amplitude (Figure 3B), suggesting that the lack of Gαi2 had some other impact on rod-mediated, but not on cone-mediated, retinal function, but the reason for this difference is not yet understood.

### The Gαi3 protein specifically interacts with the melanosomal GPCR Oa1

**Figure 4:**
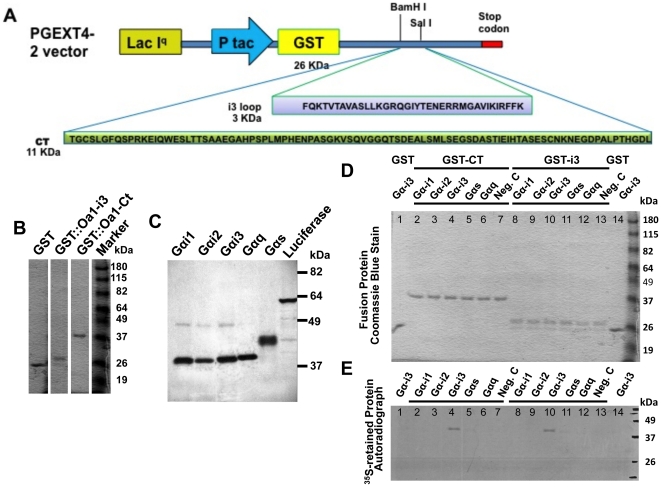
Analysis of interactions between Oa1 and heterotrimeric Gα proteins by *in- vitro* pull-down assay. A) Schematic representation of the vector used to make the GST-fusion polypeptides GST::Oa1-i3 and GST::Oa1-CT. Each amplified Oa1 sequence (i3 and CT) was cloned into the PGEXT4-2 vector containing GST between the selected restriction enzymes (BamH I and Sal I). B) Coomassie-blue stained gel showing the SDS-PAGE-separated fusion proteins used in the pull-down experiment. GST by itself has an apparent molecular weight of 26 kDa, the GST::Oa1-i3 fusion protein of 29 kDa and the GST::Oa1-CT fusion protein of 37 kDa in agreement with their estimated molecular masses. C) Autoradiogram of *in-vitro* transcribed and translated ^35^S-labeled Gα proteins that will be tested for Oa1 binding activity below (D–E), after separation by SDS-PAGE on a 10% Tris-Glycine polyacrylamide gel (D–E). The apparent molecular weight of each Gα protein is in agreement with its predicted molecular mass, Gαi1: 41 kDa, Gαi2: 40 kDa, Gαi3: 41 kDa, Gαq: 42 kDa, Gαs: 46 kDa and luciferase, run as a control: 61 kDa. **D**) Commassie blue staining of the gel used for SDS-PAGE of recombinant GST::Oa1-CT and GST::Oa1-i3 fusion proteins immobilized on glutathione-agarose beads and incubated with the indicated ^35^S-labeled Gα proteins. Lanes 1 and 14 had the GST protein incubated with one of the ^35^S-labeled Gα proteins in this experiment, ^35^S-Gαi3. Lanes 2–7: GST::Oa1-CT incubated with the indicated ^35^S-labeled Gα. Lanes 8–13: GST::Oa1-i3 incubated with the indicated ^35^S-Gα. Since the amount of ^35^S-Gα protein is minimal, only the fusion proteins are observed in this gel. Lanes 7 and 13: GST-fusion proteins immobilized on glutathione-agarose beads (Neg. C: negative control). All lanes with the same fusion protein have comparable amounts of protein. E) Autoradiograph of the same gel showing that of all ^35^S-labeled Gα proteins, Gαi3 is the only one that binds specifically to Oa1(lanes 4 and 10). Molecular weight markers are expressed in kDa.

Seven transmembrane GPCRs are characterized by their ability to couple with and activate heterotrimeric G proteins in response to a ligand binding mainly through two well-known functional regions: the third intracellular loop (i3) and the carboxy-terminal tail (CT) [8], [14]. Given that Oa1 shares all typical hallmarks of GPCRs, to characterize the potential interactions of Oa1 and Gαi3 we carried out *in-vitro* binding studies in which recombinant fusion polypeptides of glutathione S-transferase with i3 and CT regions of Oa1, immobilized on glutathione-agarose beads, were incubated with *in-vitro*-synthesized ^35^S-labeled Gα proteins. Figure 4A shows a diagram indicating how the GST fusion proteins were obtained using the PGEXT4-2 vector, as detailed in Material and Methods. Figure 4B corroborates that the apparent molecular masses of the fusion proteins, after SDS-PAGE electrophoresis, correspond to the expected 29 kDa for the GST::Oa1-i3 fusion protein and 37 kDa for the GST::Oa1–CT fusion protein; and Figure 4C shows the apparent molecular masses of the *in-vitro* synthesized ^35^S-labeled Gα proteins.

SDS-PAGE of the Oa1- Gαi interacting complexes eluted from the agarose beads and Coomassie blue staining of the gel showed that each lane contained the same amount of GST-fusion protein: lanes 2–7 and 8–13 correspond to GST::Oa1-CT and GST::Oa1-i3 reactions, respectively (Figure 4D). Interestingly, the autoradiograph of the same gel demonstrated that Gαi3 is the only one of all Gα proteins tested that specifically binds to Oa1 (Figure 4E, lanes 4 and 10). No binding was detected when GST::Oa1-i3 and GST::Oa1-CT were incubated with ^35^S-labeled Gαi1, Gαi2, Gαs, or Gαq or when GST alone, used as control, was incubated with one of the^35^S-labeled Gα proteins (we used Gαi3). Also, incubation of beads having only GST-fusion proteins (negative control) did not show any non-specific Oa1 binding with TNT rabbit retinoculocyte lysate or with the beads (Figure 4E, lanes 7 and 13).

### Mass spectrometry also identifies Gαi3 as the specific OA1-interacting Gαi protein

To identify RPE proteins that interact with human OA1 and gain a better understanding of this GPCR's function, we used a human antibody against OA1 in immunoprecipitation (IP) reactions followed by SDS-PAGE. For these experiments, we dissected the RPE from donor, adult human eyes, which allowed us to obtain sufficient amount of protein. Half of the gel was stained with Coomassie blue to visualize the protein bands. Bands in the appropriate molecular mass of Gαi proteins (39–41 kDa) and OA1 (45–48 kDa) were excised, destained, trypsinized and sent for mass spectrometry analysis to the Pasarow Mass Spectrometry core facility at UCLA. The other half of the gel was transferred to nitrocellulose membranes to confirm by Western blot analysis, using antibodies to the mass spectrometry-identified interacting protein, the specificity of the OA1 partner.

**Figure 5:**
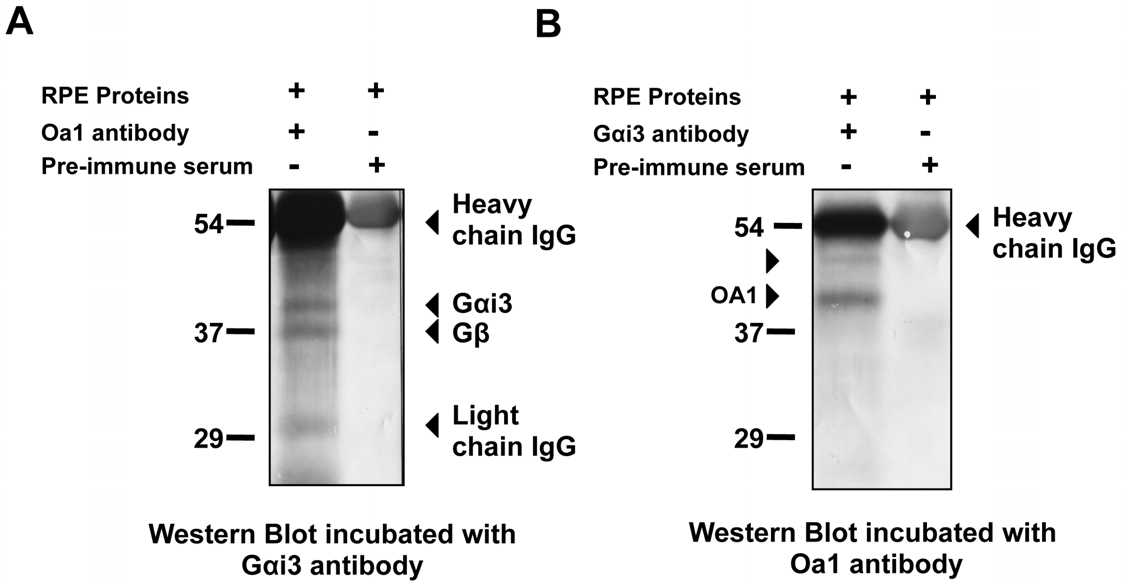
Gαi3 is the specific Oa1-interacting protein. A) Western blot of proteins from human RPE immunoprecipitated with the anti-OA1 antibody as described in Materials and Methods. SDS–PAGE was followed by incubation with anti-Gαi3 antibodies. Left lane: RPE extract incubated with OA1 antibody; right lane: RPE extract incubated with pre-immune serum. B) Western blot analysis of the proteins from human RPE immunoprecipitated with anti-Gαi3 antibody. SDS–PAGE was followed by incubation with anti-OA1 antibody. Left lane: RPE extract incubated with Gαi3 antibody; right lane: RPE extract incubated with pre-immune serum. Two isoforms of OA1 have apparent molecular weights of 45 and 48 kDa (arrowheads).

As we anticipated, mass spectrometry of the ∼38–41 kDa excised band identified Gαi3 as one of the proteins immunoprecipitated together with OA1 (Table 1). We corroborated the specific interaction of these proteins by incubating the blot with anti-Gαi3 antibody and visualizing the Gαi3 band using the ECL detection system (Figure 5A). Given that the commercially available antibodies against Gαi3 recognize both, Gαi3 and Gβ, Gβ was another protein immunoprecipitated together with OA1 and identified by mass spectrometry (Table 1). RPE lysates not immunoprecipitated with antibodies against OA1 but incubated with pre-immune serum did not show any G-protein on the corresponding lane of the blot (Figure 5A). Further confirmation of the OA1 and Gαi3 interaction was obtained by performing a reciprocal IP experiment using lysates containing proteins of adult, human RPE and the anti-Gαi3 antibody. After separating the immunoprecipitated proteins by SDS-PAGE and transferring them to nitrocellulose membranes, the blot was incubated with the anti-OA1 antibody. Our results show the presence of two forms of OA1, previously described with apparent molecular weights of 45 and 48 kDa [15], in the lane corresponding to the RPE proteins immunoprecipitated with the anti-Gαi3 antibody and the absence of the OA1 protein in the lane corresponding to the RPE lysates incubated with pre-immune serum (Figure 5B).

**Table 1:** Gαi3 is identified by LC-MS/MS as the specific Gαi protein interacting with OA1.

Accession no.	Entry	Description	Mascot score	MW	Unique peptides matched
NP_006487	Guanine nucleotide binding protein (G protein), alpha inhibiting activity polypeptide3 (Gαi-3) [Homo sapiens]	Association with Golgi-derived vesicles and protein trafficking [31]	63	41076	4
NP_0020661	G protein beta subunit [Homo sapiens]	Proteins that cover a wide variety of functions in signal transduction, pre-mRNA processing and cytoskeleton assembly [32].	234	38061	

Mascot search (Matrix Science) identified both, Gαi3 and Gβ in the immunoprecipitate of adult human RPE with the OA1 antibody. The entry names and accession numbers (UniProtKB) in this Table are provided along with a brief description of the function of the proteins and the match score, as well as the number of unique peptides found by nLC–MSMS for each protein.

### Gαi3 compensates the lack of Gαi1 in *G*α*i1-/-* mouse RPE

**Figure 6:**
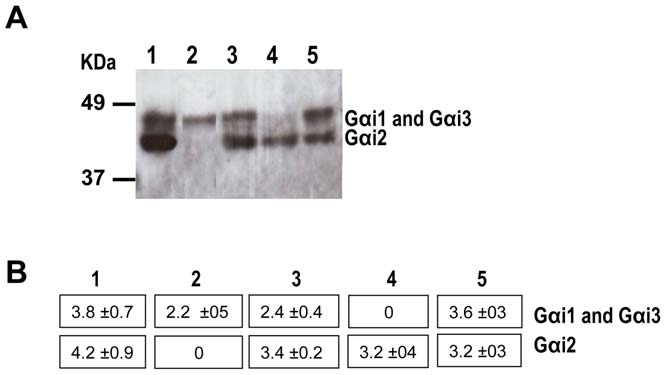
Western blot of RPE proteins from all Gαi-/- mice using an anti- Gα_common_ antibody. A) RPE membrane proteins from 3-month-old mice were separated by SDS-PAGE, blotted and reacted with Gα_common_ antibodies. Protein bands were visualized using the ECL detection reagent. The RPE proteins are from: Gαi1-/- (lane 1), Gαi2-/- (lane 2), Gαi3-/- (lane 3), DKO (lane 4) and 129 Sv (lane 5) mice. The membrane was then stripped and reacted with α-tubulin antibodies to confirm that the amount of protein loaded on each lane was the same. B) Analysis of the immunoblot by densitometry using the Quantity One 1-D Software. Numbers represent the optical density units per mm^2^ (OD U/mm^2^) of each Gαi protein band.

Compensatory increases elevating a Gαi subunit level in a tissue when another G*α*i subunit is missing from it have been previously observed [16].To test this possibility, we used Western blots incubated with Gαi_common_ antibody to analyze the expression of the three Gαi proteins in membrane preparations from RPE of *Gαi1-/-, Gαi2-/-, Gαi3-/-*, DKO and 129 Sv mice. The relative amount of protein in each band was then measured by densitometry. On the Western blot, Gαi1 and Gαi3 run together as one band at 41 kDa. In the absence of Gαi1 (in *Gαi1-/-* mice), levels of Gαi3 much higher than those in wild-type RPE are observed (Figure 6, lanes 1 and 5, respectively) since the corresponding band from the 129 Sv RPE has both G*α*i1 and G*α*i3. Densitometry measurements (Figure 6B) confirm this, showing 3.8±0.7 optical density (OD) units for G*α*i3 in lane 1 (*Gαi1-/-* RPE) and 3.6±0.3 for both G*α*i1 and G*α*i3 in lane 5 (129 Sv RPE). Gαi2 is also higher in *Gαi1-/-* RPE than in control RPE. In the case of *Gαi3-/-* mice, it is not possible to determine if a small increase in G*α*i1 is present in its RPE, since we do not exactly know how much Gαi1 or Gαi3 is present in the corresponding band from the 129 Sv RPE; the levels of G*α*i2 are comparable in these animals to those of control RPE. In contrast, absence of Gαi2 in the *Gαi2-/-* mice is accompanied by a reduced level of the 41 kDa band corresponding to the Gαi1 and Gαi3 proteins, and loss of both Gαi1 and Gαi3 in the DKO mice does not trigger an up-regulation of Gαi2 levels (Figures 6, A and B). These results suggest that at the protein level, Gαi3 may compensate completely for the loss of Gαi1 in the *Gαi1-/-* mice and that Gαi1 may compensate only partially, if it does so, for the loss of Gαi3 in *Gαi3-/-* mice.

### Gαi3 may be absent or expressed at very low levels in the RPE of *Oa1-/-* mice

**Figure 7:**
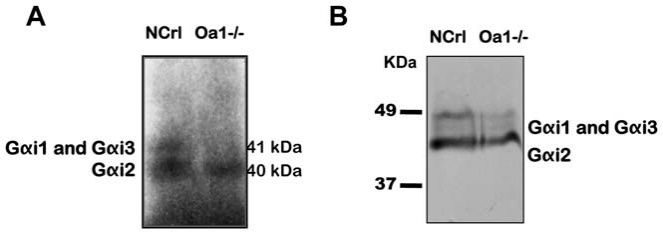
ADP-ribosylation of Gαi proteins and Western blot of *Oa1-/-* and control B6/NCrl RPE proteins. **A**) RPE membrane proteins from 3-month-old mice were subjected to ^32^P-ADP ribosylation with PTX, separated by SDS-PAGE and visualized by autoradiography. Lane 1: control B6/NCrl RPE; lane 2: *Oa1-/-* RPE. **B**) Western blot using the same RPE membrane proteins separated by SDS-PAGE and reacted with the Gα_common_ antibody. Lane 1: B6/NCrl RPE and lane 2: *Oa1-/-* RPE.

To get more insight into the Oa1 signaling cascade, we tested the functional expression of Gαi1, Gαi2 and Gαi3 in the RPE of *Oa1-/-* mice and their congenic controls, C57Bl/NCrl mice, by performing Pertussis toxin (PTX)-mediated ^32^P-ADP ribosylation experiments. After incubation of the crude membrane preparations from RPE with PTX and ^32^P-nicotinamide adenine dinucleotide (^32^P-NAD), an autoradiography of the ^32^P-labeled proteins separated on 6 M Urea-SDS-PAGE was obtained. Two bands at 40 and 41 kDa corresponding to G*α*i2 and G*α*i1 plus G*α*i3, respectively, were observed on the lane of the gel containing the RPE from control mice (Figure 7A, lane 1). In contrast, the RPE from *Oa1*-/- mice only showed the band corresponding to the ^32^P-ADP ribosylated G*α*i2. No sign of G*α*i3 or G*α*i1 was present on the autoradiograph (Figure 7A, lane 2). To corroborate these results, a Western blot was prepared with the same samples, using the Gα_common_ antibody. Figure 7B shows the G*α*i2 and G*α*i1 plus G*α*i3 bands in the control RPE (lane 1), but in *Oa1*-/- RPE, comparable levels of G*α*i2 but a very minimal amount of either or both G*α*i1 and G*α*i3 are seen (lane 2).

## Discussion

We have previously demonstrated by *in-vivo* studies on mice that the heterotrimeric G protein Gαi3 signals in the same transduction pathway controlled by Oa1 to regulate melanosomal biogenesis and axonal growth through the optic chiasm [12]. However, the interaction between Oa1 and the two other members of the Gαi family of proteins, Gαi1 and Gαi2, remained to be explored. In this paper, taking advantage of the availability of knockout mice for each of the Gαi genes, and of DKO (*Gαi1-/-, Gαi3-/-*) mice, we tested the hypothesis that Oa1 transmits its signal through one specific Gαi protein and demonstrated with *in-vitro* and *in-vivo* experiments that this protein is indeed Gαi3.

To investigate the involvement of all Gαi proteins in the regulation of size, density and shape of RPE melanosomes, electron micrographs of the RPE from 3-month-old *Gαi1-/-, Gαi2-/-, Gαi3-/-,* DKO and 129 Sv were analyzed. With regard to size, our results indicate that only the loss of function of Gαi3 significantly increases the number of macromelanosomes in the RPE. Even with loss of function of both Gαi1 and Gαi3, no added effect in the size of DKO melanosomes was observed when compared to melanosomes of *Gαi3-/-* RPE, suggesting that Gαi1 is not involved in establishing the size of RPE melanosomes. We then looked at the number of melanosomes per unit area of RPE, and found that melanosomal density in *Gαi1*-/- and *Gαi2*-/- were not significantly reduced from that in 129 Sv wild-type RPE. Conversely, melanosomal density was reduced significantly and similarly in *Gαi3-/-* and in DKO RPEs when compared to wild-type RPE. This again supports the notion that *Gαi1-/-* does not contribute to the RPE melanosomal density decrease observed in DKO mice. We also established that despite the appearance of fewer round melanosomes in *Gαi2-/-* RPEs, there is not a major change in the percent frequency of round melanosomes in all *Gαi-/-* RPEs when compared to those in the wild-type 129 Sv. In addition, as demonstrated by electroretinography, the retinas of *Gαi3-/-* as well as those of DKO mice are functional despite the phenotype of their RPEs (Figure 3A) similar to what was previously observed in the ERG of Oa1-/- mice [12]. Thus, these results together lead us to conclude that of the three heterotrimeric Gαi proteins, Gαi3 is the Oa1-associated protein involved in the regulation of RPE melanosomal size and density.

Potential binding sites for heterotrimeric G-proteins on GPCRs have been localized to the cytoplasmic loop 3 (i3) and carboxy-terminal tail (CT) of the seven-transmembrane receptors [14]. These intracellular segments are the ones that determine the interaction with specific G proteins, and as a result, which of several possible signaling pathways are activated [17], [18], [19], [20]. Therefore, we generated GST-fusion proteins with the Oa1-i3 and Oa1-CT segments and used them in GST pull-down assays to determine which Gαi protein bound *in-vitro* to Oa1. Results of these experiments showed that of the three heterotrimeric Gαi proteins, Gαi3 is the only one that binds specifically to Oa1. Most important, immunoprecipitation of RPE proteins using Oa1 antibodies, followed by SDS-PAGE and mass spectrometry analysis of the proteins, brought down Gαi3 together with Oa1, conclusively identifying Gαi3 as an Oa1-specific interacting protein. These results were further confirmed by immunoprecipitation experiments of RPE proteins using Gαi3 antibodies, which also demonstrated that Oa1 co-immunoprecipitated with Gαi3. Collectively, these data indicate that the GPCR Oa1 initiates in the RPE the signal transduction cascade that controls melanosomal size and density through activation of Gαi3.

Compensatory increases elevating a Gαi subunit level in a tissue when another Gαi subunit is missing from it have been previously observed [16]. This raises the possibility that in the RPE of Gαi knockout mice, at the protein level, compensatory expression of the other Gαi subunits may reduce the effect that the loss of a particular Gαi protein has on the control of melanosomal size and density. To test this, we measured the levels of the three Gαi proteins in the RPEs of *Gαi1-/-, Gαi2-/-, Gαi3-/-* and DKO mice using Western blotting with a Gαi_common_ antibody. Our results suggest that at the protein level, only the loss of Gαi1 from the RPE may be compensated for by Gαi3. However, with respect to functional compensation of the melanosomal phenotype, we cannot conclude that Gαi3 functionally compensates for the loss of Gαi1 since our results show that *Gαi1-/-* melanosomes are not significantly different from those observed in control mice, even though they are a bit larger. Similarly, the fact that the DKO phenotype is no worse than that of *Gαi3-/-* alone with respect to size, density or shape of RPE melanosomes, suggests that Gαi1 is not critical for the regulation of these parameters. Furthermore, compensatory increases, if any, in Gαi1 protein in the *G*α*i3-/-* retina seem to play no role in melanosome biogenesis. Although Gαi2 levels are considerably increased in the *Gαi1-/-* animals and to a quite lesser extent in *Gαi3* knockouts, we have shown in the *in vivo* experiments that G*α*i2 is not involved in the determination of melanosome size. Given the high levels of this protein in the RPE, it must play an important, though different, role in this tissue than G*α*i3, a role having quite discriminable effects, evidenced in the ERG itself (Figure 3B).

ADP-ribosylation of Gα subunits is a covalent modification catalyzed by PTX in which an ADP-ribose moiety is attached to the C-terminus of the protein. This prevents the Gi proteins to interact with their receptors and, therefore, it causes functional inactivation of all signal transduction pathways. ADP-ribosylation with NAD ^32^P-labeled on its ADP-ribose moiety allowed us to tag Gαi subunits in membranes and to learn about their involvement in cellular responses. We carried out this reaction using RPE membranes of *Oa1-/-* and their congenic B6/NCrl mice. Interestingly, our results showed that the three G*α*i subunits are present in the RPE of control mice, but that only G*α*i2 is in the *Oa1*-/- RPE. Similarly, when we used samples from the same RPE membranes to perform Western blotting with the Gα_common_ antibody we found that Giα2 levels in -/- Oa1 RPE are comparable to those in the control RPE, but that there is very minimal, if any, amount of either or both G*α*i1 and G*α*i3 in the *Oa1-/-* RPE. These interesting results will lead to a whole series of studies in the future.

It is well established that in addition to their important roles in many pathways of transmembrane signaling, where they participate in processing and sorting of incoming signals as well as in adjusting the sensitivity of the signaling system, heterotrimeric G- proteins are also localized to the Golgi complex [21], where they are involved in the formation of secretory vesicles from the trans-Golgi network (TGN) [9]. Gαi3, in particular, acts as an inhibitor of intra-Golgi and post-Golgi trafficking [22], and has been found, among other places, in the membranes of secretory vesicles in pancreatic acinar cells, from where it facilitates the fusion between zymogen granules and/or the expulsion of vesicular contents [23].

The specific function of G*α*i3 in the RPE has not been identified. Our working hypothesis is that after the bulk of Oa1, together with tyrosinase, TRP1 and other compounds involved in melanin synthesis, has reached the stage II melanosomes, Oa1 starts to activate G*α*i3, which in turn inhibits the traffic of vesicles carrying the membrane proteins required for melanization from the TGN. However, melanization occurs with no problem in stage III and IV melanosomes, as they use the proteins already present. This would explain why the levels of Oa1 decrease from those in stage II melanosomes to those in stages III and IV [24]. Oa1 is not being renewed in the melanosomes after its activation of G*α*i3 because there are no longer vesicles bringing this protein to the melanosomes. Thus, the function of G*α*i3 in the RPE would be to control the size of melanosomes through the inhibition of vesicle trafficking from the TGN to the melanosome, a function previously thought to be carried by Oa1 [25].

**Figure 8:**
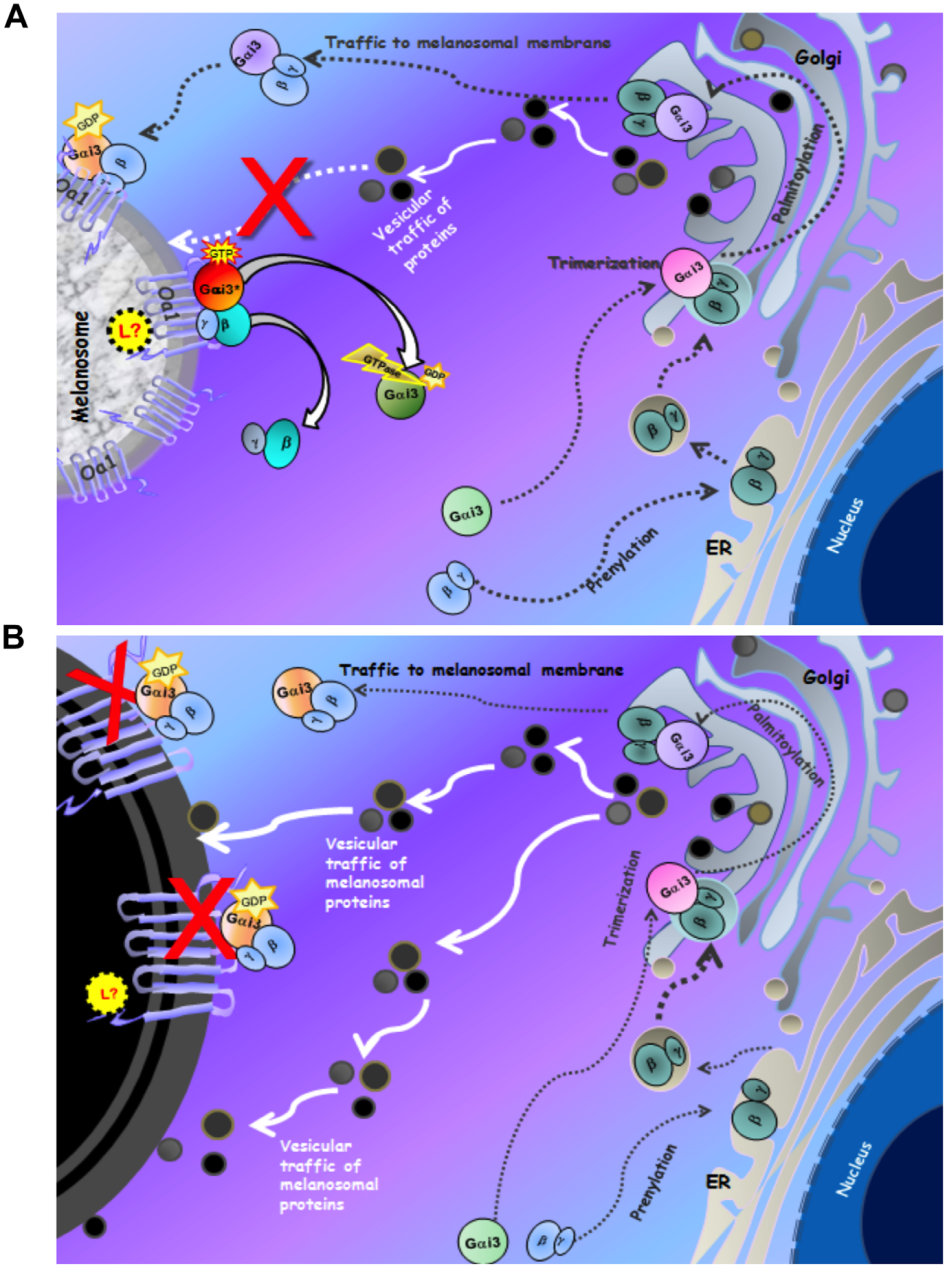
Proposed hypothetical model of the G*α*i3 regulation of RPE melanosomal size. A) An unknown luminal ligand (L?) turns on wild-type Oa1, which activates Gαi3. The active Gαi3 in turn inhibits vesicular traffic of proteins from the TGN to the melanosome, controlling in this way its size. B) Mutated OA1 is unable to activate Gαi3 and, thus, the continuous supply of melanin-related proteins to the melanosome results in the formation of very big organelles, the macromelanosomes.

On the basis of our results and the accumulated information in the literature about GPCRs, Gαi3 functions and melanogenesis, we are proposing a hypothetical model for the beginning of the Oa1 signaling cascade (Figure 8A).

In this model, at the end of stage II melanogenesis, when melanosomes have acquired their melanogenic proteins and the bulk of Oa1, a signal must turn on an endogenous lumenal ligand to activate Oa1 at the melanosomal surface membrane. Like in other GPCR cascades, activated Oa1 will cause the exchange of GDP for GTP on the Gαi3 subunit of the heterotrimeric G protein localized to the surface of the same membrane, activating Gαi3 (Gαi3*) –which then separates from the βγ subunits– and leading to the release of Gαi3* and Gβ**γ** to the RPE cytoplasm. Gαi3* will at this stage inhibit the vesicular traffic of membrane proteins from the TGN to the melanosome. Prenylation of the Gγ subunit will target the Gβγ complex to the endoplasmic reticulum, where it will be processed fully before it is delivered to the Golgi. There, Gβγ will bind Gαi3 and after post-translational modification of Gαi3, the G protein heterotrimer may leave the TGN and get transported to the melanosome surface membrane using the classic secretory pathway.

This model could also explain the presence of macromelanosomes in the RPE of ocular albinism patients or *Oa1* and *G*α*i3*-/- mice (Figure 8B). Mutations in the *OA1* gene in humans could render the OA1 protein incapable of activating the heterotrimeric Gαi3 on the surface membrane of the melanosome. The same effect would be observed in the absence of Oa1 or Gαi3 in knockout mice. Without the active form of Gαi3*, inhibition of the vesicular traffic of melanin-related proteins to stage II melanosomes cannot occur, and the continuous supply of this material to the melanosome would result in the formation of abnormally large organelles, the macromelanosomes.

In summary, while the precise function of Gαi3 in RPE melanosome biogenesis remains to be delineated, it is clear that this protein plays an important role in the control of the size of RPE melanosomes. As a consequence, Gαi3 is also controlling RPE pigmentation, which seems to be necessary during embryonic stages for the proper decussating pattern of the optic axons. Because *G*α*i3-/-* mice, like *Tyr-/-* and *Oa1-/-* mice, all show abnormalities in the decussation of their optic axons [12], Gαi3 appears to be the common effector by which these three distinct RPE phenotypes affect the retinal ganglion cells as their axons navigate the optic chiasm.

## Materials and Methods

### Ethics Statement

All experiments involving mice were carried out using protocols approved by the UCLA Animal Research Committee, and in accordance with the ARVO Statement for Use of Animals in Ophthalmic and Vision Research.

### Animal and human tissues

C57BL/6NCrl (B6/NCrl) mice and congenic *Oa1* knock-out mice (*Oa1-/-*) were obtained from The Charles River Labs, USA and Italy, respectively, and bred at UCLA. G*αi1-/-*, G*αi2-/-*, G*αi3-/-* and the DKO mice were previously generated on the 129 Sv background. The genotype of these mice was determined by Southern blot analysis of mouse tail genomic DNA as described by Jiang et al. [26]. Mice were housed and bred in conventional cages and environmental conditions at the animal facilities of UCLA.

Healthy human donor eyes were obtained from the National Disease Research Interchange (Philadelphia, PA) and immediately frozen in liquid nitrogen. The donor eyes were handled in compliance with the Declaration of Helsinki.

### Electron Microscopy

3 month-old 129 Sv, G*αi1-/-*, G*αi2-/-, Gαi3-/-* and DKO mice were deeply anesthetized by an intraperitoneal injection of 120 mg/kg sodium pentobarbital, and perfused intracardially with 2% paraformaldehyde and 2.5% glutaraldehyde in 0.1 M sodium phosphate buffer, pH 7.4. Eyes were enucleated, rinsed in 0.1 M phosphate buffer, post-fixed with 1% buffered osmium tetroxide, dehydrated in graded ethanol, and embedded in araldite 502. Sections for electron microscopy (60–70 nm) were cut on a Leica Ultracut UCT and collected on 200 mesh uncoated copper grids. For the ultrastructural analysis, stained sections (5% uranyl acetate and 0.4% lead citrate) were observed with a 910 Zeiss electron microscope. The RPE fields analyzed were photographed using a Keenview™ digital camera. For quantification of melanosomes and determination of their area in the RPE, we analyzed 50 micrographs for each 129 Sv and DKO mice and 30 micrographs for each *Gαi1-/-*, *Gαi2-/-* and *Gαi3-/-* mice at 16,000× magnification using the analySIS™ software for LEO 900 TEM, version 3.2. (Soft Imaging System, Lakewood, CO). Images were cropped with Adobe Photoshop (Adobe Systems Inc., San Jose, CA). A total of 2501 129 Sv, 1285 *Gαi1-/-*, 1609 G*αi2-/-*,1115 *Gαi3-/-* and 1997 DKO melanosomes were studied, sampling 10 eyes for each 129 Sv and DKO mice and 6 eyes for each *Gαi1-/-*, *Gαi2-/-* and *Gαi3-/-* mice. The data for the *Gai3-/-* mice were previously reported, but are included herein for direct comparison.

#### Melanosomal size and density

We used the magic wand feature of the Soft Imaging System analySIS software to select and measure the area of every individual melanosome and of the total RPE area containing the melanosomes in each micrograph, as we have done previously in the analyses of *Gαi3-/-* and the *Oa1-/-* mice and their corresponding controls [12]. We analyzed the difference in the melanosomal size, the percent frequency of melanosomes in the >5000 nm^2^ size range, and the number of melanosomes per RPE area among five mouse groups: 129 Sv, *Gαi1-/-*, *Gαi2-/-* and *Gαi3-/-* and DKO. Comparisons among all groups were done using a one way analysis of variance (ANOVA) followed by post-hocTukey tests to identify significant differences between individual pairs of groups, conservatively using animal averages for each measure and thus an n of 5 for the 129 Sv and DKO groups and an n of 3 for the *Gαi1-/-*, *Gαi2-/-* and *Gαi3-/-* groups. A p-value of less than 0.05 was considered to be statistically significant.

#### Melanosomal Morphology

To determine melanosomal shape we used the particle detection analysis of the Soft Imaging System analySIS software for LEO 900 TEM, version 3.2. The classification of the shape of the organelles is given by how round the organelle is. A melanosome with a shape factor of 1 is round, while elliptical melanosomes have a shape factor less than 1. We compared the percentage of round melanosomes for each particular mouse line. The differences among all mouse groups [129 Sv, *Gαi1-/-*, *Gαi2-/-*, *Gαi3-/-* and DKO] were compared using one-way ANOVA and post-hoc Tukey tests, all as above.

### Electroretinography

Mice were anesthetized with an intraperitoneal injection of xylazine (0.5 mg/ml) and ketamine (1 mg/ml) in normal saline. In adult mice, a dose of 0.1 ml was administered. Body temperature was maintained at 38°C with a heating pad. Pupils were dilated with Atropine (1%). A gold-wire electrode was placed on the corneal surface of the right eye and referenced to a gold wire in the mouth. A needle electrode in the tail served as the ground. Responses were amplified (Tecktronix AM 502 Differential Amplifier, ×10,000) band pass filtered (0.1–300 Hz), digitized using an I/O board (PCI-6221, National Instruments, Austin, TX) in a personal computer, and averaged. A signal rejection window was used to eliminate electrical artifacts. All stimuli were presented in a large integrating sphere painted with a highly reflective white matte paint (#6080, Eastman Kodak Corporation, Rochester, NY). Rod mediated responses were obtained with blue flashes (Wratten 47A; I_max_ = 470 nm) varied over an intensity range of 3.5 log units. Cone-mediated responses were obtained with white flashes on a rod saturating background (32 cd/m^2^).

### Production of GST::OA1-i3 and GST::OA1-CT recombinant proteins

Mouse Oa1 cDNA was subcloned into the mammalian expression vector pCDNA3.1/V5-His-Topo using EcoRI and PstI sites in the polylinker. Using this construct, a 108 bp DNA fragment corresponding to the third cytosolic loop (i3) of OA1 (residues 213–248), with flanking fragments corresponding to the BamHI and SalI restriction enzymes, was amplified by PCR using the following primers: Forward, 5′-TGGA TCCTTTCACAAGACAGTGACTTCA-3′; and Reverse, 5′-AGTCGACTCATTTG AAAAAACGGGTCTTGAT-3′. Similarly, a 275 bp DNA fragment corresponding to the C terminal (CT) of OA1 (residues 314–405), with flanking fragments corresponding to the BamHI and SalI restriction enzymes, was amplified by PCR using the following primers: Forward, 5′-TGGATCCACAGGATGCAGCCTGGATGTC-3′; and Reverse, 5′-AGTCGACTCAGAGTTCCCCCTGGGCTTGGGA-3′. The 25 µl reaction contained 2.5 µg of each 5′ primer and 3′ primer, 100 ng/µl pCDNA3.1/V5-His-Topo–OA1, 0.6 Units of Taq DNA polymerase (Invitrogen Corporation, Carlsbad, CA), 1× PCR buffer, 2.5 mM MgCl_2_ and 250 µM dNTP mix. After 30 cycles (melting for 3 min at 94°C; annealing for 1.5 min at 53°C; extension for 1 min at 72°C), the PCR reactions were subjected to an additional 5 min incubation at 72°C. Each PCR reaction yielded a single product that was subsequently isolated and gel purified, cut with BamHI and SalI, and subcloned into the pGEX-4T-2 vector (Amersham Bioscience, now GE Healthcare, Piscataway, NJ) using T4 ligase. Ligation reactions were used for transformation of BL21 (DE3) *E. coli*, and were selected on LB-ampicillin agar plates. Positive clones were screened by restriction analysis and DNA sequencing.

### Preparation of GST-Fusion Proteins

*E. coli* cultures carrying the pGEX-4T-2 constructs were grown until they reached mid-logarithmic growth phase (0.4–0.5 *A*_600_). Isopropyl-β-D-thiogalactopyranoside (IPTG) was then added to a final concentration of 0.1 mM to induce GST protein expression, and the cultures were further incubated for 4 h at 30°C. GST was purified from cultures incubated at 37°C for 3 h. The GST::Oa1-i3 fusion protein was obtained as previously described [27] with few modifications. Briefly, bacteria were harvested by centrifugation at 4,000 *g* for 15 min and the pellets were resuspended in 2 ml of STE Buffer (10 mM Tris, pH 8.0, 150 mM NaCl, 1 mM EDTA, 25 µg/ml PMSF and protease inhibitors cocktail) containing 50 µg/ml of lysozyme (added immediately prior to resuspension), and incubated on ice for 15 min. Dithiothreitol (DTT) was then added to a final concentration of 2.5 mM. Bacteria were lysed by the addition of 1.0% N-lauroylsarcosine (Sarkosyl), disrupted on ice for 1 min using a MISONIX Sonicator 3000 (power level 3, 50% cycle) and centrifuged at 13,000 *g* for 10 min at 4°C to remove insoluble debris. To obtain the GST::Oa1-CT fusion protein, a similar procedure was followed, but no lysozyme or Sarkosyl was used.

### Purification of GST-tagged proteins

GST-tagged proteins were purified from bacterial lysates by affinity chromatography using immobilized Glutathione Sepharose™ High Performance (GE Healthcare). 1 ml GSTrap HP column was equilibrated with 5 ml of binding buffer (PBS, pH 7.3) and 1 ml of bacterial lysate was loaded onto it. The column was washed with 5 ml of binding buffer and the GST-tagged protein was eluted with 3 ml of elution buffer (50 mM Tris-HCL, 10 mM reduced glutathione, pH 8.0).

### GST activity assay

The GST specific activity was measured spectrophotometrically using the GST assay kit (Sigma-Aldrich Corporation, St. Louis, MO) according to the manufacturers' instructions, using 1 mM 1-chloro-2,4-dinitrobenzene (CDNB) and 2 mM reduced-glutathione (GSH) as substrates at 340 nm and 25°C. Fractions with good GST activity (∼600–800 µg/µl) were used for incubations with *in-vitro* transcribed/translated ^35^S-methionine-labeled Gα proteins (see below).

### I*n-vitro* synthesis and ^35^S-methionine-labeling of Gα Proteins

Gαi1, Gαi2, Gαi3, Gαs and Gαq were i*n-vitro* transcribed, translated and labeled with ^35^S-methionine using the pAGA-2 vector containing the cDNA of the corresponding Gα protein and the TNT Coupled Reticulocyte Lysate system (Promega, Madison, WI) with T7 RNA polymerase. The resulting ^35^S-labeled proteins (1.5 µg of each) were boiled in 2× SDS–PAGE sample buffer containing 3% β-mercaptanol, loaded onto a 10% Tris-Glycine gel and electrophoresed at 45 V until the dye had run out of the gel. After washing with water for 10 min (3 times), and immersing in 5% glycerol for 15 min, the gel was dried and visualized by autoradiography. The relative expression of ^35^S-labeled Gα proteins was analyzed by densitometric measurement using Quantity One 1-D analysis software (version 4.4, Bio-Rad, Hercules, and CA). The mean density for each band, expressed as number of pixels, was used to calculate the ratio of each protein to the ^35^S-labeled Gα protein that had the lowest number of pixels (ratio = 1). This number was used to determine the appropriate volume of each ^35^S-labeled Gα protein that was used for the GST-pull down assay.

### GST-pull-down assays

For *in-vitro* protein-protein interaction assays, 10 µl of each ^35^S- Gα-protein expressed from the pAGA-2 vector containing the same amount of protein were incubated with 50 µl of Immobilized Glutathione agarose resin (Thermo Fisher Scientific Inc., Waltham, MA), ∼1 µg of GST protein or the GST-fusion protein (GST::Oa1-i3 or GST::Oa1-CT), and 100 µl of Binding buffer (50 mM Tris, pH 7.5, 100 mM NaCl, 0.5% Triton 100×) for 2 h at room temperature (RT). The beads were recovered by centrifugation at 3,000 g, 4°C, for 1 min, washed for 5 min (5 times) with 500 µl of ice-cold Binding buffer, and centrifuged again for 1 min at 8,000 g. The protein-protein interacting complex was eluted from the beads with 30 µl of 2× SDS–PAGE sample buffer containing 3% β-mercaptanol and its components were separated by SDS-PAGE as above, on a 10% Tris-glycine gel. The gel was fixed and Coomassie blue-stained to confirm that the fusion proteins were present in comparable amounts and had the right molecular weight. After destaining, the gel was dried and exposed to X-ray film overnight (−80°C) to visualize the interacting ^35^S-Gα-proteins.

### Mass Spectrometry Analysis

Several steps were followed to prepare samples for mass spectrometry:

#### Immunoprecipitation and Western Blotting

Normal human RPE was dissected from frozen donor human eyes. Tissue was homogenized on ice with a Dounce homogenizer in 500 µl RIPA lysis buffer containing 50 mM Tris–HCl pH 7.5, 150 mM NaCl, 0.5% Na-deoxycholate, 0.1% SDS, 1 mM PMSF, 1× complete protease inhibitor cocktail (Roche Molecular Biochemicals, Indianapolis, Indiana), and 5 mM N-ethylmaleimide (Sigma-Aldrich Corporation). Homogenates were pre-cleared with 100 µl of protein G–Sepharose (Thermo Fisher Scientific, Inc.) for 1 h at 4°C. Lysates (500 µl) were first incubated with 17 µl of anti-OA1 (ABCAM, Cambridge, MA) or with 17 µl anti-Gαi3 (Santa Cruz Biotechnology, Inc., Santa Cruz, CA) rabbit polyclonal primary antibodies, at 4°C overnight, and then with 50 µl of protein G–Sepharose for 2 h at RT. The complex-bound resin was washed 5 times with IP buffer (25 mM Tris–HCl, 150 mM NaCl; pH 7.2) and 3 times with water. Immunoprecipitated complexes were eluted with 2× SDS–PAGE sample buffer containing 3% β-mercaptanol and each of duplicate aliquots were loaded onto a different half of a 12% Tris-glycine gel. SDS-PAGE was carried out overnight at 44 V. Half of the gel was stained with Coomassie blue to visualize protein bands for mass spectrophotometry analysis and the other half was transferred to a nitrocellulose membrane (Hybond ECL, Amersham Biosciences) overnight at 33 V. The blot was then incubated with 1∶10,000 anti-Gαi3 or anti-OA1 rabbit primary antibodies, and with 1∶10,000 goat anti-rabbit secondary antibodies. The Enhanced Chemiluminescence (ECL) detection reagent (Amersham Biosciences) was used to visualize the bands.

#### Tryptic Digestion

Protein bands of interest on the Coomassie blue–stained gel were excised and placed in microtubes. In-gel digestion with trypsin was performed according to standard procedures routinely used in the Pasarow Mass Spectrometry core facility at UCLA. Briefly, the gel pieces were distained with 10 µl acetonitrile (ACN) for 30 min at RT and treated with 10 mM DTT/100 mM NH_4_HCO_3_ (200 µl) for 1 hour at 37°C. Samples were then alkylated with 55 mM iodoacetamide (Sigma-Aldrich Corporation). The gel pieces were washed with 100 mM NH_4_HCO_3_ for 15 min at RT, dehydrated with ACN and digested with 1.25 µg trypsin (Promega) in 100 µl of 50 mM NH_4_HCO_3_, on ice, for 45 min. They were then incubated overnight in 10 µl of 50 mM NH_4_HCO_3_, without trypsin. The digests were extracted twice with 100 µl of 50% ACN/0.5% formic acid at RT for 60 min with constant mixing, the extracts were pooled and dried and each sample was then reconstituted with 8 µl of 2% formic acid and sent for nano-liquid chromatography tandem mass spectrometry (nLC-MSMS) analysis at the Pasarow core facility.

#### Database Analysis

The mass spectra were searched against a human trypsin indexed database, with variable modifications of carboxyamidomethylation, methionine oxidation, and deamination of asparagine residues using the Bioworks software (Thermo Fisher) based on the SEQUEST algorithm implemented in Discoverer software (Thermo Fisher). Spectra were also searched using Mascot software (Matrix Science, UK) and results with p<0.05 (95% confidence interval) were considered significant and indicating identity.

### Preparation of RPE Membranes

The RPEs from *Gαi1-/-, Gαi2-/-, Gαi3-/-*, DKO, *Oa1-/-* and their corresponding 129 Sv and B6/NCrl control mice (12 eyes of each), were dissected, collected and frozen in liquid nitrogen. Each sample was homogenized in 250 µl of 27% (wt/wt) sucrose/1 mM EDTA/10 mM Tris·HCl, pH 7.5, in Dounce homogenizers. Homogenates were centrifuged for 5 min at 1,000 g and the supernatants were again centrifuged at 12,000 g for 20 min to obtain the RPE membranes. Quantification of protein from melanin-containing RPE membranes was carried out by the method of Sedmak and Grossberg, which depends on the conversion of Coomassie brilliant blue G-250, in diluted acid, from a brownish-orange to an intense blue color [28]. With this very sensitive method the melanin interference is negligible. The G-250 dye was prepared as a 0.06% solution in 19% perchloric acid (w/v) and was filtered through Whatman No. 1 filter paper to remove any undissolved material. The assay consisted of adding 0.5 ml of the G-250 dye to 5 µl of homogenized RPE membranes, mixing immediately, and determining absorbance at 620 nm.

### Western blot analysis

The Gαi1, Gαi2, and Gαi3 protein levels in RPE membranes from 129 Sv, *Gαi1-/-, Gαi2-/-, Gαi3-/-* and DKO mice, were measured using an anti-Gα_common_ antibody (Cell Signaling technology, Danvers, MA) that recognizes all the Gαi and Gαo proteins. Proteins (15 µg per lane) were separated by SDS-PAGE as above on 9% acrylamide/bis-acrylamide gels containing 6 M urea and blotted onto nitrocellulose. Blots were incubated with 1∶2,000 anti-Gα_common_ polyclonal anti-rabbit primary antibody, overnight, at 4°C and with 1∶5,000 goat anti-rabbit secondary antibody for 3 hours at RT. The ECL detection reagents were used to visualize the bands.

### ADP ribosylation of Gαi proteins

Using the membrane proteins from B6/NCrl and *Oa1-/-* mice we performed ADP-ribosylation as described in Jiang et al. [29], [30]. The reaction mixture (30 µl) contained 15 mM Tris-HCl, pH 8.0, 1 mM EDTA, 10 mM thymidine, 1 mM ATP, 1 mM guanosine 5′-0-(2-thiodiphosphate) (GDPβS), 2 mM dithiothreitol, 0.17% Lubrol PX, 0.02% bovine serum albumin, 10 µg/ml PTX activated by DTT (50 mM), 5×10^6^ cpm ^32^P-NAD (∼2×10^6^ M) and 15 µg protein of crude RPE membranes. The mixture was incubated at 4°C overnight. The reaction was stopped by addition of 10 µl of complete 2× Laemmli's sample buffer containing 9% β-mercaptoethanol and 4 mM unlabeled NAD. The ADP-ribosylated proteins were separated by high resolution urea-SDS-PAGE (9% acrylamide/bis-acrylamide gel containing 6 M urea), overnight, at 44 V. The gel was dried and autoradiographed. Expected bands were observed at 41 *kDa* for the Gαi1 and Gαi3 and at 40 *kDa* for the Gαi2 ribosylated proteins.
